# A genome-wide assessment of the population structure of thirteen admixed and pure Australian beef cattle breeds

**DOI:** 10.1093/jas/skag242

**Published:** 2026-08-10

**Authors:** Zeinab Manzari, Natalie K Connors, Julius H J van der Werf, David J Johnston, Mohammad H Ferdosi

**Affiliations:** AGBU, A Joint Venture of the NSW Department of Primary Industries and Regional Development, University of New England, Armidale, New South Wales 2351, Australia; AGBU, A Joint Venture of the NSW Department of Primary Industries and Regional Development, University of New England, Armidale, New South Wales 2351, Australia; School of Environmental and Rural Science, University of New England, Armidale, New South Wales 2351, Australia; AGBU, A Joint Venture of the NSW Department of Primary Industries and Regional Development, University of New England, Armidale, New South Wales 2351, Australia; AGBU, A Joint Venture of the NSW Department of Primary Industries and Regional Development, University of New England, Armidale, New South Wales 2351, Australia

**Keywords:** Australia, beef cattle, genomic population structure, admixed breeds, pure breeds

## Abstract

Knowledge of population structure is a key factor for successful multi-breed genomic prediction, especially in single-step analysis when metafounders are considered. In Australia, current assessments mostly focus on single breeds using a single-step genomic prediction method. However, the effective integration of pedigree, phenotypic, and genomic data in a multi-breed framework still requires further research, especially for combined analyses including admixed and multi-breed populations. This study began with 602,952 genotyped individuals with 8K SNPs in common from 13 beef cattle breeds (Alexandria, Angus, Brahman, Brangus, Charolais, Droughtmaster, Hereford, Kynuna, Limousin, Santa Gertrudis, Shorthorn, Speckle Park, and Wagyu). Due to different numbers of animals being genotyped in each breed, a representative subset of animals was chosen by employing a validated sampling strategy using Gaussian Mixture Models (GMM) complemented by Principal Component Analysis (PCA) within each breed. Subsequently, a specific number of animals in each cluster were randomly selected to capture the entire genetic diversity per breed, with a total of 260 animals from each breed. The first three principal components explained 59.89% of the total variation, with PC1 (33.54%) clearly separating *Bos indicus* from *Bos taurus* lineages. Admixture analysis identified stable ancestral components and defined the genetic makeup of both pure and composite populations. The results showed extensive genetic diversity in some breeds and highlighted distinct genetic differences between *Bos indicus* and *Bos taurus* breeds. In addition, six composite breeds’ admixture levels confirmed their origin and breed history, revealing a directional shift in ancestry proportions by a longitudinal increase in Brahman ancestry within tropical composites over time. Thus, the findings pave the way for more effective utilization of genetic diversity both within and across populations and provide a framework for designing multi-breed genetic evaluations and breeding programs to improve productivity and profitability in Australian beef production.

## Teaser

This study presents a comprehensive analysis of the genomic population structure in 13 Australian beef cattle breeds, including both admixed and pure breeds, to identify genetic relationships among them that can inform the development of more informed cattle breeding strategies.

## Introduction

Australia’s beef industry plays a key role in the national economy. Australia is a major beef exporter, ranking among the top global suppliers (Read 2024). Approximately 45% of Australia’s landmass is located in a tropical environment (Turton 2017), and raising beef cattle is the main form of land utilization in this environment. *Bos indicus* (or Zebu) cattle breeds have genetic adaptations suitable for tropical locations because they are resistant to harsh climatic conditions such as drought, heat, and humidity, and challenging environmental conditions such as limited water, poor pasture quality, parasites, and diseases (Rubio-Lozano et al. 2021). Although *Bos indicus* cattle are well-adapted to these environments, they typically produce less beef and lower meat quality compared to *Bos taurus* breeds. Consequently, crossbreeding between *Bos taurus* breeds with superior production traits and *Bos indicus* breeds with tropical adaptability has been implemented as an effective strategy to enhance beef production in Northern Australia (Burrow 2015). Generally, the greater the percentage of *Bos indicus* in the crossbreds, the more adaptation to tropical climates. Conversely, a greater *Bos taurus* content indicates higher productivity. As a result, these Australian admixed beef cattle breeds were developed to improve beef production, especially in tropical environments. The diversity of the Australian beef cattle results from the sequential introduction of British, Continental European, Japanese, and Indian genetics over the past two centuries. These foundational lineages led to the development of admixed breeds, such as the Alexandria and Kynuna, which were designed to maintain heterozygosity levels while improving adaptation to tropical environments, a challenging objective in relatively small closed populations and enhancing fertility and meat quality traits (McDonald 2018). The Alexandria composite was designed to contain 12.5% Africander, 37.5% Brahman, 12.5% Charolais, 6.25% Hereford, and 31.25% Shorthorn (Figure S1). The Kynuna composite was designed to contain 12.5% Brahman and 37.5% Shorthorn, and 25% of both Red Angus and Tuli (contains approximately 20% *Bos indicus*; McDonald 2018; Ling et al. 2025; Figure S1).

The breakthrough in affordable genotyping technologies has revolutionized the understanding of the impacts of selection on genetic variation and the evolution of cattle breeds (Matukumalli et al. 2009). Since the mid-1980s, the BREEDPLAN genetic evaluation system has been developed to improve economically important traits. The BREEDPLAN is Australia’s national beef genetic evaluation system and has been applied to almost 40 breeds (Crook et al. 2019). Connors et al. (2017) suggest that BREEDPLAN currently uses single-step genomic prediction; this method combines pedigree, phenotypic, and genotype data to estimate the genetic merits of each animal. However, the industry is increasingly moving toward multi-breed genomic evaluation, which requires comprehensive knowledge of population structure and breeding history across breeds. That is, where pedigree information is incomplete or missing, multi-breed genomic prediction can be achieved by aligning the pedigree-based relationship matrix (NRM) with genomic relationship matrix (GRM). This alignment is accomplished by adjusting allele frequencies to reflect the population’s genetic structure, either using breed-specific frequencies or applying a Q matrix representing breed composition (Legarra et al. 2015; Anglhuber et al. 2024). In addition, metafounders can be used to partially replace traditional genetic groupings, thus offering better representation of population structure for the pedigree and improving GRM and NRM alignment. Such an approach requires knowledge of the genetic composition of individuals within each breed (including assessing their genetic similarities) as well as genetic distances and historical relationships between breeds to establish accurate modeling of the allele frequencies to construct the GRM and to build the gamma matrix used in metafounder methods. Thus, this research aims to address these issues by understanding the current population structure of Australian beef cattle breeds and the genetic makeup of the admixed breeds that have been formed over the last several decades. These findings provide a framework for improving breeding programs through accurate multi-breed predictions, leading to enhanced efficiency and profitability.

## Material and methods

### Population history

This study analyzed the genomic population structure of 13 Australian beef cattle breeds. These beef breeds were classified into two groups: pure breeds, including the Angus, Brahman, Charolais, Hereford, Limousin, Shorthorn, and Wagyu; and admixed populations, including Alexandria, Brangus, Droughtmaster, Kynuna, Santa Gertrudis, and Speckle Park. Purebred animals were from *Bos indicus* and/or *Bos taurus* lineages.

To obtain a general overview of the genomic architecture of the populations studied, it is necessary to consider the timeline of their formation or introduction to Australia. The Australian beef industry is built upon a foundation of British, Continental European, Japanese, and Indian lineages. The Angus was the first to be established, arriving in Tasmania in 1824 (Angus Australia 2026), followed by the Hereford in 1826 (Herefords Australia 2023). Continental European breeds were introduced later, including the Charolais, which arrived via semen in 1969 (Charolais Society of Australia 2026), and the Limousin, which became established during the 1970s (Australian Limousin Breeders’ Society Ltd 2026). The genomic landscape was further expanded by the importation of tropically adapted cattle. This transition was driven by the importation of Brahman cattle by the Council of Scientific and Industrial Research (later CSIRO) in 1933, with subsequent large-scale expansion enabled by significant US bloodline imports from 1982 onwards (Australian Brahman Breeders Association 2026). Finally, the Wagyu breed represents a more recent introduction, with foundational Japanese genetics acquired between the late 1980s and 1990s (Australian Wagyu Association 2026).

The introduction of indicine genetics served as the basis for several admixed breeds, specifically developed to enhance environmental resilience in northern Australia. The Droughtmaster was pioneered in North Queensland in the early 1900s after initial Zebu imports in 1910 (Droughtmaster Australia 2026), while Brangus cross-breeding originated in the 1930s before becoming a stabilized breed in 1951 (BrAngus Australia 2026b). The Santa Gertrudis was introduced from the United States in 1952, marking the first major tropically adapted importation to Australia (Santa Gertrudis Breeders Association of Australia 2026). More recently, the North Australian Pastoral Company (NAPCo) developed closed-herd composites, including the Alexandria (established 1982–1986) and the Kynuna (developed in the mid-1990s) to balance fertility and meat quality (McDonald 2018). Notably, while most Australian admixed breeds are indicine-influenced, the Speckle Park represents a distinct category. Introduced from Canada in 2007, the Speckle Park is an admixed breed without Brahman background, and it has seen rapid expansion due to its superior carcass quality (Speckle Park International 2026).

### Genotype data

The genomic data were extracted from databases provided for the BREEDPLAN evaluation and filtered using the BREEDPLAN genomic pipeline quality control criteria (Connors et al. 2017). Genotyping of animals spanned multiple decades, with purebred animals (Angus and Hereford) being genotyped over 40 yr, while admixed breeds were genotyped more recently, primarily within the last 20 yr (Figure S2). This timeline reflects the long-term history of breed development and selection in Australia.

Each breed was genotyped using different SNP chip densities, ranging from 7K to 800K SNPs. A common SNP density for all genotyped animals was constructed. This procedure was performed separately per breed. Initially, SNPs were merged into a consolidated panel, though arrays with greater than 160K density were excluded due to the limited number of individuals genotyped at those densities. Missing SNPs were imputed and the haplotype library was reconstructed using FImpute v3 with pedigree (Sargolzaei et al. 2014), as explained by Connors et al. (2017). Subsequently, newly genotyped animals were iteratively imputed using both a haplotype library and pedigree records, with phased genotypes being continuously added to the haplotype library to support future iterations. To ensure marker reliability, only SNPs with a missingness rate of less than 90% were retained, as markers with a greater missingness rate may compromise imputation accuracy. After imputation, genotype data from all 13 beef cattle breeds were merged, and around 8K SNPs common to all breeds were identified. In this study, only autosomal chromosomes were considered. Table 1 presents the initial SNP density of each breed before common SNPs and the sample size of each breed.

**Table 1:** Sample descriptions for different beef cattle breeds used.

Breed	Abbreviation	Sample^a^	SNP density	PCs^b^	Clusters^c^
**Alexandria**	xa	11,553	112,481	9	13
**Angus**	aa	292,816	82,728	7	13
**Brahman**	bb	48,380	64,322	4	10
**Brangus**	bg	8,226	44,627	5	10
**Charolais**	cc	5,734	62,052	7	10
**Droughtmaster**	dm	7,674	61,762	8	13
**Hereford**	hh	59,388	82,892	6	13
**Kynuna**	xk	4,909	52,620	8	13
**Limousin**	ll	3,810	58,377	7	10
**Santa Gertrudis**	sg	8,244	100,542	8	13
**Shorthorn**	ss	328	33,451	8	5
**Speckle Park**	sk	13,143	81,336	7	13
**Wagyu**	wy	138,747	82,753	6	13

^a^Sample: Number of genotyped animals; ^b^PCs: Number of principal components for clustering; ^c^Clusters: Number of genetic clusters.

### Identifying subsets within breeds

Principal component analysis (PCA) is the most common strategy for reducing the dimensionality of variables (ie, SNPs) while retaining as much of the variability in the dataset as possible (Jolliffe and Cadima 2016). In population structure analysis, it is necessary to select an equal number of individuals of each breed, especially in PCA, to avoid bias due to differences in sample sizes. Therefore, subsetting animals based on genetic clusters within each population is an effective way to equalize sample sizes and ensure uniform representation of the entire population in the analysis. For example, if there are influential sires in the population with a substantial number of offspring, selecting random individuals will result in more offspring of these sires. Therefore, genotype clustering and sub-setting within breeds were performed in several steps:

In the first step, to explore the divergence within breeds, twenty principal components (PCs) were calculated using PLINK v2 (Purcell et al. 2007) with the “—pca approx 20” command for all genotyped animals from those breeds with more than 5,000 individuals and “—pca 20” for breeds with less than 5,000 animals to explain more genetic variation and better segregation of populations. Galinsky et al. (2016) explained that the “—pca approx” command applies random matrix theory to approximate PCs, and more significantly reduces computational time and resources for larger data sets than does the “—pca” command. In this study, the variance of each PC was displayed in a scree plot to highlight the relative contribution of each component in explaining the genetic structure in the data. Density scatterplots were generated using the “ggplot2” R package to visualize genetic dispersion along the first two principal components (PC1 and PC2) (Wickham et al. 2016). We selected PCs cumulatively explaining >50% of variance because this threshold captures the major axes of genetic variation while reducing noise from minor components. This approach ensures that selected PCs contain enough information about the genetic structure of the breed to perform subsequent clustering. The second step in the process was the application of the Bayesian Information Criterion (BIC) to the results obtained for the selected principal components to determine the optimal number of clusters for each breed (Fraley et al. 2012). These BIC curves consistently followed an asymptotic pattern across all populations, rising sharply before leveling off. This strategy captures the true genetic structure while avoiding over-fitting (Scrucca et al. 2016). The Gaussian mixture model (GMM) was then employed to cluster individuals within each breed, and for each cluster identified in the previous step. The GMM algorithm clustered variables based on different characteristics of the geometric distribution, that is, orientation, shape, and volume (Scrucca et al. 2016). Like the BIC results, the “mclust” R package (Fraley et al. 2012) was used to complete GMM calculations.

The final step of the process required random selection of a specified number of animals from each cluster; selecting representatives from each genetic subgroup (cluster) avoids potential bias due to unbalanced sampling. For example, in a breed with ten identified clusters, 26 animals were selected per cluster, resulting in a total of 260 for the breed. This number (260) was determined on the basis of the smallest population size among the breeds (Shorthorn) and then employed for all breeds.

#### Validation of subsampling strategy and SNP panel independence

Multiple independent validation approaches were employed to ensure that each 260-animal subset accurately represented the genetic diversity of the full population while minimizing sampling bias and confirming marker independence within the SNP panel.

Genetic Diversity and Differentiation: Multi-locus heterozygosity (MLH) and fixation index (F_ST_) were compared between the full population and the 260-animal subset using the common 8K SNP panel. MLH was calculated using the formula (N − O)/N, where N is the number of non-missing genotypes and O is the number of observed homozygous genotypes, using PLINK’s “—het” command. F_ST_ values were also computed using Weir and Cockerham’s estimator in PLINK.

Replication Stability: The random sampling process within each cluster was repeated three times independently to assess the robustness of the stratified clustering approach. The PCA and ADMIXTURE analysis based on supervised mode (*K* = 7) were re-run on each replicate subset.

Linkage Disequilibrium (LD): LD decay was calculated across all 13 breeds using PLINK to validate that the 8K common SNP panel provided sufficient marker independence. Pairwise *r*² values were computed between all SNP pairs as a function of physical distance (kb) for each breed independently.

Pedigree-Based Paternal Diversity: The paternal representation of our 260-animal subsets was investigated. For each breed, the total genotyped population served as the reference population. We identified the ten most influential sires within each reference population based on total progeny count and compared their cumulative representation against our sampled subsets to ensure the 260-animal group was not over-represented by dominant industrial lineages. To further quantify paternal lineages, we calculated the progeny-per-sire ratio by dividing the matched subset size (*n*_matched_) by the number of unique sires (Table S3). The *n*_matched_ value represents the count of individuals in the 260-animal subset that were identified with a valid, non-zero paternal record in the pedigree data. This ensures that the calculation only accounts for animals with known ancestry; individuals with missing records (Sire ID = 0) were excluded. Unique sires refer to the total number of distinct paternal IDs identified within the subset, where each sire is counted only once regardless of how many offspring he has in the sample. All breeds had pedigree records, except for Shorthorn and Wagyu.

### Population structure across beef cattle breeds

#### Principal component analysis

PLINK (parameter*—*pca 20) was used to examine data from all subsets of 260 animals to examine segregation patterns and genetic relationships across different populations (both within and across breeds). The first two PC distributions were plotted using “ggplot2” R package (Wickham et al. 2016).

#### Phylogenetic relationships

Two methods were used to examine phylogenetic relationships among breeds. Firstly, the pairwise fixation index (F_ST_) (Weir and Cockerham 1984) was applied to measure and compare genetic differences between breeds and depicted in a matrix as a heatmap and dendrogram, using the “pheatmap” R package (Kolde and Kolde 2015). The F_ST_ measures genetic distance between populations. This measurement describes genetic differentiation based on the variance of allele frequencies between populations relative to the total variance in the entire population (Holsinger and Weir 2009). F_ST_ values range from 0 (minimal genetic differentiation) to 1 (greater differentiation) between breeds. Secondly, the pairwise genetic distances were analyzed using PLINK v2 with the command “—distance square 1-ibs.” This allows for the neighbor-joining (NJ) phylogenetic tree to be employed using the “ape” R package (Paradis and Schliep 2019). These phylogenetic relationship analyses (F_ST_ and NJ) aim to evaluate the genetic distance between breeds and to identify how admixed breeds descend from other breeds.

#### Breed composition of admixed breeds

Individual ancestry proportions and shared ancestral components were estimated for the six composite and seven purebred populations using ADMIXTURE v1.3.0 (Alexander et al. 2009). An unsupervised analysis was initially performed with *K* values ranging from 1 to 20 to determine the optimal number of ancestral clusters (K). The optimal *K* was identified using a 5-fold cross-validation (CV) procedure, where the *K* value exhibiting the greatest stability and an ‘elbow’ shape in the CV error curve was selected for downstream interpretation.

After the unsupervised assessment, a supervised ADMIXTURE analysis was executed at *K* = 7, utilizing the seven purebred populations (Angus, Brahman, Charolais, Hereford, Limousin, Shorthorn, and Wagyu) as reference ancestors. This allowed for the precise quantification of progenitor contributions within the admixed populations. Visualization of all ancestry profiles was generated using the “pophelper” R package (Francis 2017). Moreover, the supervised run was merged with birth year data to evaluate genomic stability and investigate potential directional shift in ancestry proportions. For the five tropical admixed breeds (Alexandria, Brangus, Droughtmaster, Santa Gertrudis, and Kynuna), a linear regression model was applied to estimate the annual rate of change in mean Brahman ancestry over time.

## Results and discussion

### Within-breed genetic architecture

The PCA conducted separately for all populations to explore the internal genetic structure of each breed. The variance explained by the first two components (PC1 and PC2) is provided through PCA density-scatter plots (Figure S3). These plots revealed that some breeds do not uniformly dominate the genetic space but instead form different discrete density concentrations. In contrast to some highly centralized purebreds (Angus, Charolais, and Wagyu), other breeds, such as the Brahman and Santa Gertrudis, displayed two to three high-density concentrations. These concentrations may reflect the temporal distribution of genotyping; as shown in Figure S2, there was a sharp increase in genotyping starting from 2010, with a significant surge between 2015 and 2022 or potentially a divergence between traditional lineages. Therefore, different factors are also likely to contribute to the formation of different discrete density concentrations within breeds. Moreover, Srikanth et al. (2024) interpreted these concentrations as the result of selection over time and the disproportionate influence of specific elite sire lineages. This is supported by the Santa Gertrudis population data presented in Table S3, where the top ten sires account for 12.19% of all genotyped individuals.

A similar complexity was observed in the Brahman, three density concentrations observed in the Brahman plot (Figure S3) are due to the long history of breed formation and genotyping or multiple foundational bloodlines imported into Australia at different stages. Koufariotis et al. (2018) found that Brahman is strongly influenced by *Bos indicus* genetics, with only around 10% influence by *Bos taurus*, which may explain the structural differences in the genomic data in this study. Brahman also exhibited the highest PC1 explained variance at 26.33, nearly double the levels seen in pure *Bos taurus* breeds such as the Angus (13.59) or Hereford (15.08).

Brangus displayed a diffuse genomic cloud, unlike the tight clustering observed in its parental Angus and Brahman populations. The Brangus maintained an intermediate level of structural diversity (PC1: 23.73%). This diffuse pattern reflects the ongoing segregation of the founding *Bos taurus* and *Bos indicus* genomes due to the Australian registry rules, indicating that while the Brangus is a recognized breed, it maintains a diverse range of progenitor ancestry. In Australia, Brangus cattle are allowed to have between 25% and 75% Brahman blood (Brangus Australia 2026a). This regulatory flexibility accounts for the diffuse pattern observed in the within-breed PCA (Figure S3), confirming that the breed’s genomic structure is strongly shaped by these registry standards.

### Clustering analysis of genetic subsets within breeds

The PCA-complemented GMM successfully identified the internal genetic structure of each breed, allowing us to build a balanced 260-animal subset. We selected PCs that collectively explained over 50% of the genetic variance to ensure that the clustering was based on the most significant genetic differences within each population (Table 1, Figure S4). The optimal number of clusters varied among breeds. Smaller or more uniform populations, such as the Shorthorn, required only five clusters to capture their genetic structure. In contrast, larger and more diverse breeds required between 10 and/or 13 clusters (Table 1, Figure S5). This variation shows that the method was flexible enough to handle the different levels of complexity across the 13 breeds. Selecting an equal number of animals from each cluster served as an effective strategy to equalize sample sizes while maintaining genetic diversity.

#### Validation of breed-representative subsets and SNP panel informational content

Several validation analyses were performed to verify that the representative subset provided a robust characterization of the full populations and SNP independence.

Firstly, genetic differentiation between the full populations and the 260-animal subsets was evaluated using Weir and Cockerham’s F_ST_. The F_ST_ values were negligible across all 13 breeds (Table S1), indicating almost zero or even negative genetic divergence and confirming that the subsampled animals accurately captured the genetic architecture of their full populations without introducing sampling bias. The phylogenetic relationships derived from the 260-animal subsets (Figure 2) had minor differences with those calculated from the full populations (Figure S6) due to different sample sizes.

Secondly, the MLH was evaluated to compare genetic diversity across the groups. The MLH for the 260-animal subset was nearly identical to the full population when utilizing the same common SNP panel 0.405 (260-animal subset) vs 0.406 (the full population) in Angus (Table S2). However, a divergence was observed when comparing the common SNP to the original density in the Brahman population (0.277 [the full population with common SNP] vs 0.390 [the full population with original SNP density]; Table S2). Despite this change in values for the Brahman, the diversity remained stable across all breeds. This difference in marker informativity for the Brahman is supported by Boichard et al. (2012), who reported that while taurine dairy and beef breeds maintain high polymorphism (∼98%), the Brahman breed often exhibits a reduced proportion of polymorphic loci (89.7%) on lower-density due to ascertainment bias. Additionally, Pérez O’Brien et al. (2014) and McTavish et al. (2013) demonstrated that SNP selection often favors markers with greater polymorphism in *Bos taurus* lineages, which leads to a reduction of diversity values in indicine breeds.

Thirdly, the random sampling within each cluster was performed three times independently to ensure the subsampling strategy was unbiased. Comparison of the resulting PCA and ADMIXTURE across these three iterations revealed no deviations between results, confirming that the observed population structures were stable (Figures S7 and S8).

Fourthly, pedigree-based analysis also validated that the PCA-complemented GMM approach successfully prioritized paternal diversity. Across the study, the subsets comprised a high number of independent paternal lineages (mean = 171 unique sires; Table S3). The Hereford subset exhibited the highest paternal diversity with 222 unique sires and a progeny-per-sire ratio of 1.17, indicating that the sample is composed of single-progeny sires. In contrast, the Kynuna subset showed the lowest unique sire count (105) due to incomplete pedigree records (*n*_matched_ = 212) rather than a lack of genetic diversity. While the representation of the top 10 most influential sires was proportionally higher in the 260-animal subsets than in the full populations in some admixed breeds (Table S3). These dominant sires are the key genetic contributors to the contemporary breed. All these influential individuals must be included in the subset to show the true genetic identity of each breed. The results of this method also minimized the risk of contemporary family bias. These validation methods provide high confidence that the identified population structures reflect long-term historical development and prevent influential sires with many offspring from dominating the data, which often happens with simple random sampling.

Finally, the analysis of LD decay confirmed that the common 8K SNP panel provided sufficient marker independence across all 13 breeds (Figure S9). Pairwise *r*^2^ values dropped below the 0.2 threshold rapidly, typically within 100–200 kb. While the British breeds (Angus and Shorthorn) exhibited higher LD, the Brahman and its admixed breeds showed a more rapid decay. The divergence in LD patterns between subspecies has been widely reported (Pérez O’Brien et al. 2014; Chishi et al. 2025). This rapid decay in our indicine-influenced populations also reflects a degree of ascertainment bias, consistent with the reduction in Brahman MLH observed in this study (Table S2). The LD findings showed that the markers capture independent variation and do not affect the population structure analyses.

### Genomic population structure and breed composition across breeds

By selecting a representative subset from each breed, as outlined above, we were able to investigate the genetic population structure of all 13 Australian beef cattle breeds and perform a comprehensive analysis of interbreed relationships.

#### Principal component analysis across breeds

**Figure 1:**
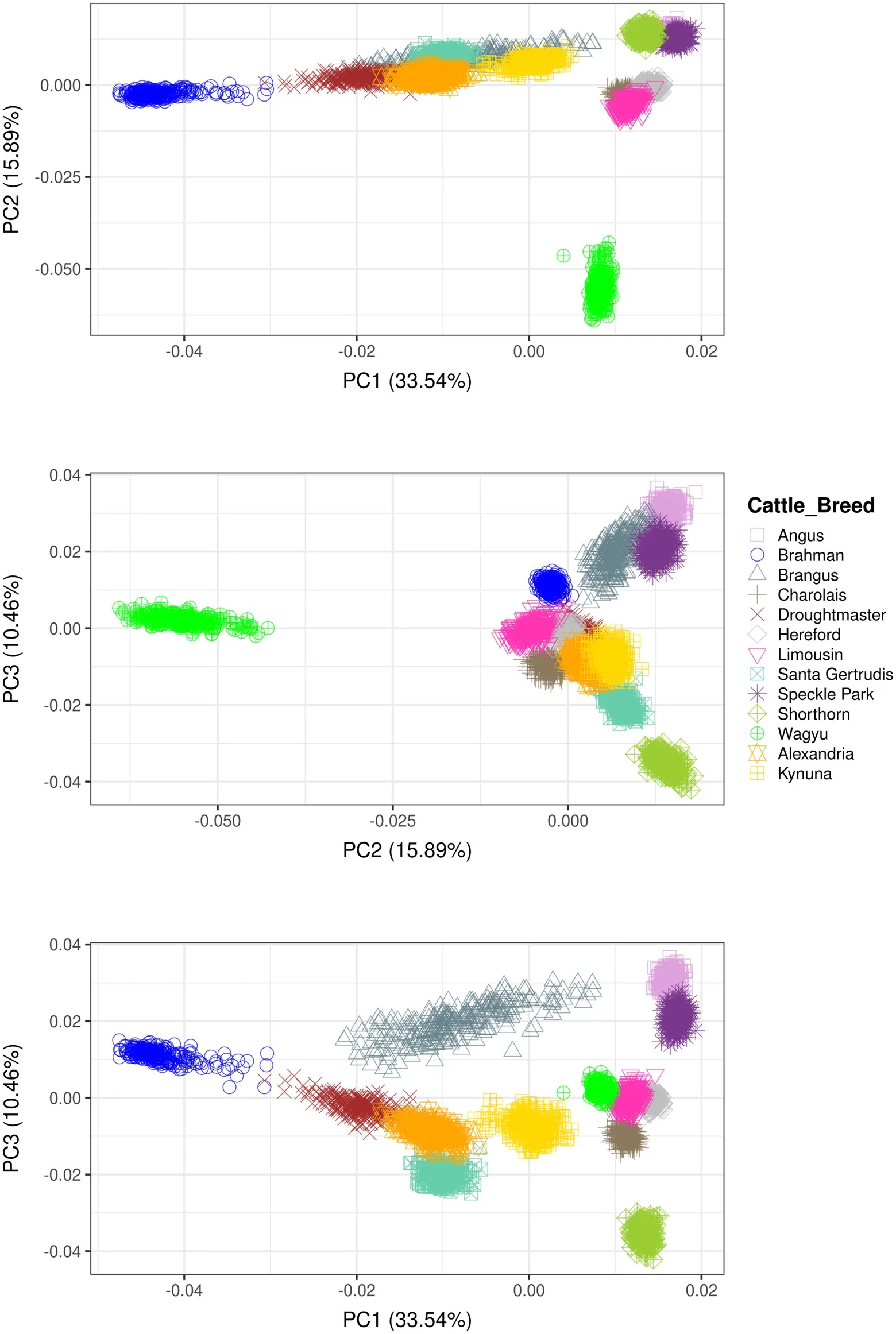
A plot of the first three principal components (PC1, PC2, and PC3) for 13 Australian beef cattle breeds.

The PCA of the common genomic intersection across 13 populations revealed a clear genetic architectural separation driven by ancestral origin and geographical development. The first three principal components accounted for a substantial proportion of the total genomic variation, with PC1, PC2, and PC3 explaining 33.54, 15.89, and 10.46%, respectively (Figure 1). The PC1 captured the deep genetic divergence between *Bos* *taurus* and *Bos* *indicus*, a pattern consistent with the global cattle structure reported by McTavish et al. (2013) and Decker et al. (2014). The triangular patterns in the three corners of the PC1 clearly distinguish between *Bos* *indicus* and *Bos* *taurus* breeds. The second and third components further resolved within-subspecies diversity, specifically distinguishing the Wagyu via its distinct Asian taurine background and highlighting the genomic uniqueness of the Shorthorn and Angus lineages.

The admixed breeds were located in intermediate positions between their shared ancestries, which confirms their hybrid heritage and reflects the crossing of subspecies to combine environmental resilience with carcass quality. While all five admixed breeds were grouped toward the Brahman to varying degrees, their specific positions reflect the relative strength of the indicine contribution to each population. More specifically, Droughtmaster was located closer to the Brahman than those for other admixed breeds, indicating a higher proportional genetic contribution from *Bos indicus* genetics. This finding is consistent with research by Lamb (2023), who investigated the population structure of Australian beef cattle and identified a higher Brahman contribution in the Droughtmaster compared to Santa Gertrudis and other tropical composites. The PCA patterns and distinct intermediate positioning of these populations suggest they have reached a high degree of genomic stability since their formation. Paim et al. (2020) reported that while admixed breeds begin as intermediate placeholders between their progenitors, they eventually diverge to form their own unique clusters. The Brangus population showed a more diffuse genomic cloud compared to other breeds due to a wide range of parental ancestry.

#### Phylogenetic relationships

**Figure 2:**
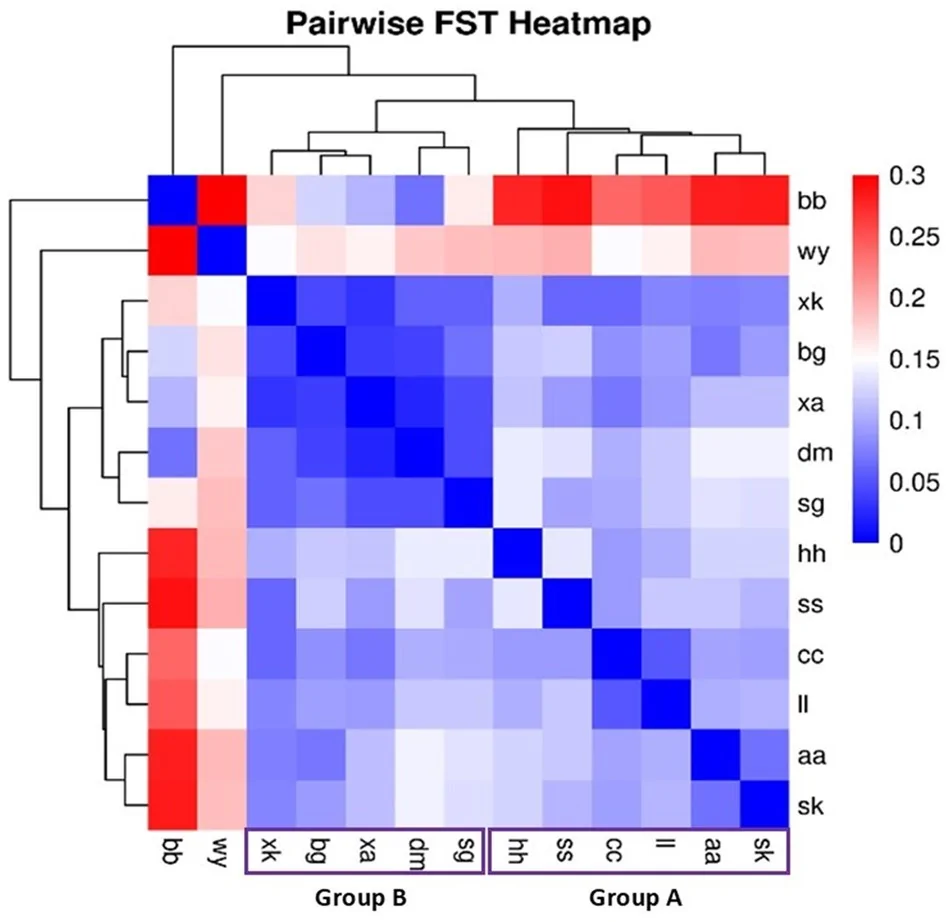
Phylogenetic relationships for 13 Australian beef cattle breeds. The heatmap and dendrogram of pairwise fixation index (F_ST_). aa, Angus; bb, Brahman; bg, Brangus; cc, Charolais; dm, Droughtmaster; hh, Hereford; ll, Limousin; sg, Santa Gertrudis; sk, Speckle Park; ss, Shorthorn; wy, Wagyu; xa, Alexandria; xk, Kynuna.

The phylogenetic relationships between breeds were examined to investigate genetic divergence and evolutionary history. The pairwise F_ST_ values for the breeds are presented in a heatmap and dendrogram (Figure 2). The dendrogram showed several distinct sections, including two main groups (Group A, all *Bos* *taurus* breeds; Group B, all admixed breeds with a background of the *Bos* *indicus*) as well as two long branches for *Bos* *indicus* (Brahman) and East Asian taurus (Wagyu) breeds. This structure represents the genetic differences between breeds and the degree of genetic proximity or distance between them. Additionally, breeds originating from the same branch indicated a closer genetic relationship than those from longer branches, which suggests that breeds with long branches diverged a long time ago.

Within Group A (Figure 2), the two French continental breeds (Charolais and Limousin) were positioned on short, shared branches, indicating low genetic differentiation. Similarly, the Speckle Park clustered tightly with the Angus, displaying a closer genetic relationship to this temperate ancestor than to the Shorthorn. This finding highlights the significant impact of the Angus genome on contemporary Australian Speckle Park; a pattern also reflected in the PCA results (Figure 1). In contrast, the Hereford and Shorthorn were located in more distinct positions. The separate branch for the Hereford is consistent with the findings of Decker et al. (2014), which identified Hereford cattle as forming a distinct northwestern European breed that diverged earlier than many other modern European breeds.

Group B (Figure 2) consisted of populations with stabilized *Bos indicus* introgressions. These admixed breeds exhibited the lowest mutual genetic distances, likely due to their derivation from similar founding events using shared ancestors. For instance, the short branch distance between the Droughtmaster and Santa Gertrudis is attributed to their common Brahman and Shorthorn bloodlines. The Alexandria, Brangus, and Kynuna further underscore the Brahman genetic background of these admixed breeds. As expected, the Brahman and Wagyu breeds exhibited the longest external branches, confirming their high degree of genetic distinctiveness and unique evolutionary trajectories. The heatmap further supported the indicine bloodline within the admixed breeds. The Brahman displayed lower genetic divergence from the Droughtmaster than from the Santa Gertrudis. These results suggest that the Droughtmaster may preserve a marginally higher proportional genetic contribution from the Brahman compared to the Santa Gertrudis, an observation supported by the PCA positioning. As Australian breed standards specify that the Droughtmaster was developed with a 3/8 to 1/2 Brahman contribution, and the Santa Gertrudis foundational standard is 3/8 Brahman, our genomic results are consistent with these progenitor proportions after decades of selection.

Finally, the NJ tree based on the Identity-By-State (IBS) distance matrix (Figure S10) provided a fine-scale view of genetic distance. The admixed breeds with the highest indicine contributions (Droughtmaster) were positioned closest to the Brahman branch, while those with lower introgressions (Kynuna) were more distantly placed. The extended branch lengths observed for the Brahman and Brangus populations likely reflect higher levels of within-breed genetic distance compared to other breeds.

#### Genomic ancestry and breed composition of Australian composites

**Figure 3:**
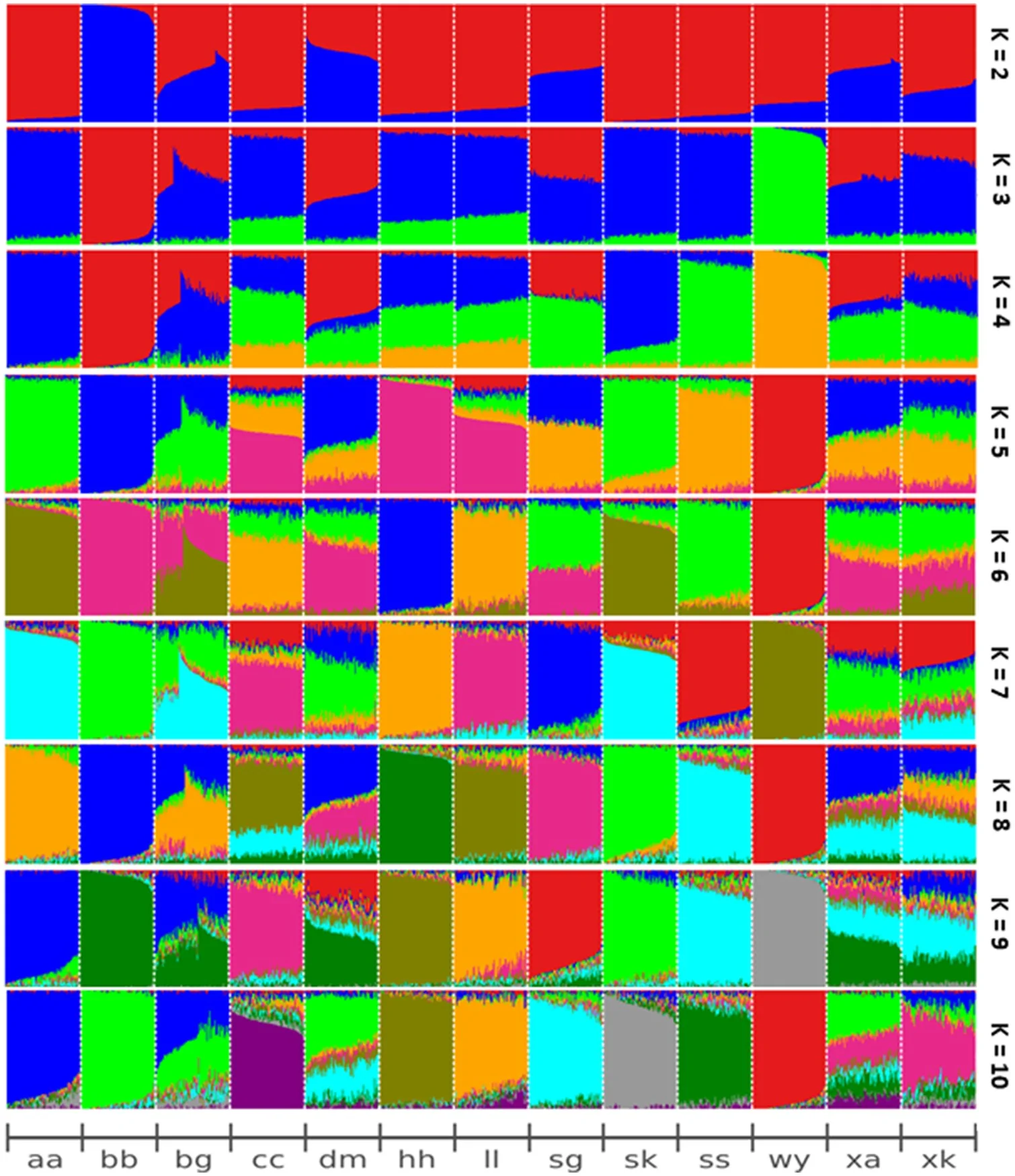
The unsupervised ADMIXTURE analysis of 13 Australian beef cattle breeds with bar plots for the clusters of *K* = 2 to *K* = 10. aa, Angus; bb, Brahman; bg, Brangus; cc, Charolais; dm, Droughtmaster; hh, Hereford; ll, Limousin; sg, Santa Gertrudis; sk, Speckle Park; ss, Shorthorn; wy, Wagyu; xa, Alexandria; xk, Kynuna.

Two admixture analyses were conducted to understand the patterns of genetic ancestries within admixed and pure breeds. Firstly, the ADMIXTURE was analyzed in an unsupervised mode on the genomic data from a set of unrelated individuals (without knowledge from reference populations), illustrating the distribution of genomic contributions across different breeds (Figure 3). The optimal number of ancestral clusters (K) was evaluated through unsupervised CV from *K* = 1 to *K* = 20. As shown in Figure S11, the CV error follows a sharp downward until *K* = 7, after which the curve becomes asymptotic. This supports the observation that while higher K values (up to *K* = 20) technically provide lower error, they likely capture within-breed genetic drift or specific family structures rather than broad ancestral signatures, consistent with findings by Lawson et al. (2018). Additionally, in unsupervised mode, the stability of results decreases with increasing *K*. Under these situations, individuals with the true content of ancestry are low, and estimates are not only uninterpretable but also often change unpredictably (Alexander and Lange 2011). In Figure 3, each vertical line in the bar plot indicates a single animal using different colors showing the amount of the estimated admixture from the ancestral breed. The unsupervised mode of ADMIXTURE software depends on using target population-specific information to allocate proportions of ancestry (Strucken et al. 2021). Consequently, the specific genetic makeup of the target populations influences the admixture estimates, complicating the direct comparison of ancestral proportions across different breeds. Although unsupervised admixture analyses do not precisely aim to identify the ancestral breed composition, they do illustrate genetic diversity among the breeds derived from *Bos* *indicus* and *Bos* *taurus* species. Gobena et al. (2018) reported that the unsupervised mode is susceptible to influences from sources of population structure beyond heterogeneous breed ancestry; without defined breed-specific allele frequencies, familial relationships within the sample can bias allele frequency estimates. For ancestral clusters *K* = 2–10, admixed cattle breeds demonstrated a mixture with other breeds, while some pure breeds exhibited negligible diversity with other breeds.

**Figure 4:**
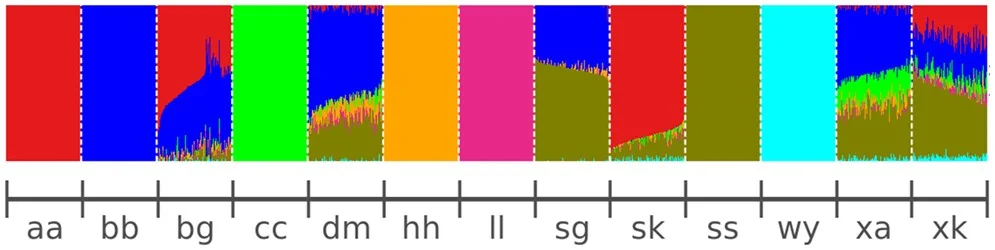
The supervised ADMIXTURE analysis of 13 Australian beef cattle breeds with bar plots for clusters of *K* = 7. aa, Angus; bb, Brahman; bg, Brangus; cc, Charolais; dm, Droughtmaster; hh, Hereford; ll, Limousin; sg, Santa Gertrudis; sk, Speckle Park; ss, Shorthorn; wy, Wagyu; xa, Alexandria; xk, Kynuna.

Secondly, an admixture analysis was performed based on a supervised mode analysis with *K* = 7, being the number of pure breeds in the study (Figure 4). Australian admixed beef cattle breeds revealed variable admixture proportions from Asian, European, and Indian breeds. Comparing admixed breeds with purebreds is essential for several reasons, including accurately estimating the genetic breed composition, adjusting the allele frequencies according to each animal’s genetic breed composition, assessing the genetic distance between breeds, and improving predictive accuracy in multi-breed genetic models, especially in crossbreed populations. All admixed breeds presented diverse degrees of breed composition according to their genetic structure and breeding history. The two NAPCo admixed breeds (Alexandria and Kynuna) had genetic background contributions from various ancestral components (Figure 4), which generally differed from other admixed breeds. For example, Alexandria demonstrated a shared genetic background with many different breeds that were its ancestors and included in this study, such as Brahman, Charolais, Hereford, and Shorthorn, strongly supporting its admixture with these breeds during the crossbreeding program (Figure S1). The Brangus breed displayed evidence of admixture exclusively between Angus and Brahman, while also maintaining a high level of genetic diversity within the breed. This genetic diversity was confirmed by the results of PCA (Figure 1) and phylogenetic tree analysis (Figure S10). Both the Droughtmaster and Santa Gertrudis were admixed with Brahman and Shorthorn. However, the Droughtmaster had a little more admixture from some of the European cattle breeds (Figure 4). In addition, ADMIXTURE results, along with PCA and phylogenetic relationships, support that Droughtmaster has more Brahman content than Santa Gertrudis. Speckle Park was the only admixed breed in this study with exclusively *Bos taurus* lineages. It shared a large proportion of ancestry with Angus and a smaller portion with Shorthorn, which was also supported by PCA and dendrogram results to confirm its temperate genomic architecture.

The genetic background of Angus contributed more to Speckle Park, Brangus, and Kynuna, respectively. In addition, the genetic contribution of Brahman was higher in Droughtmaster, Brangus, Santa Gertrudis, Alexandria, and Kynuna, respectively. Similarly, the Shorthorn genetic background in the admixed breeds was higher in Santa Gertrudis, Kynuna, Alexandria, Droughtmaster, and Speckle Park, respectively, according to their genetic background. The systematic factors in the crossbreeding strategy and random factors as a result of Mendelian sampling during gametogenesis could be key factors influencing the different percentages of ancestor content in each breed (Kelleher et al. 2017).

**Figure 5:**
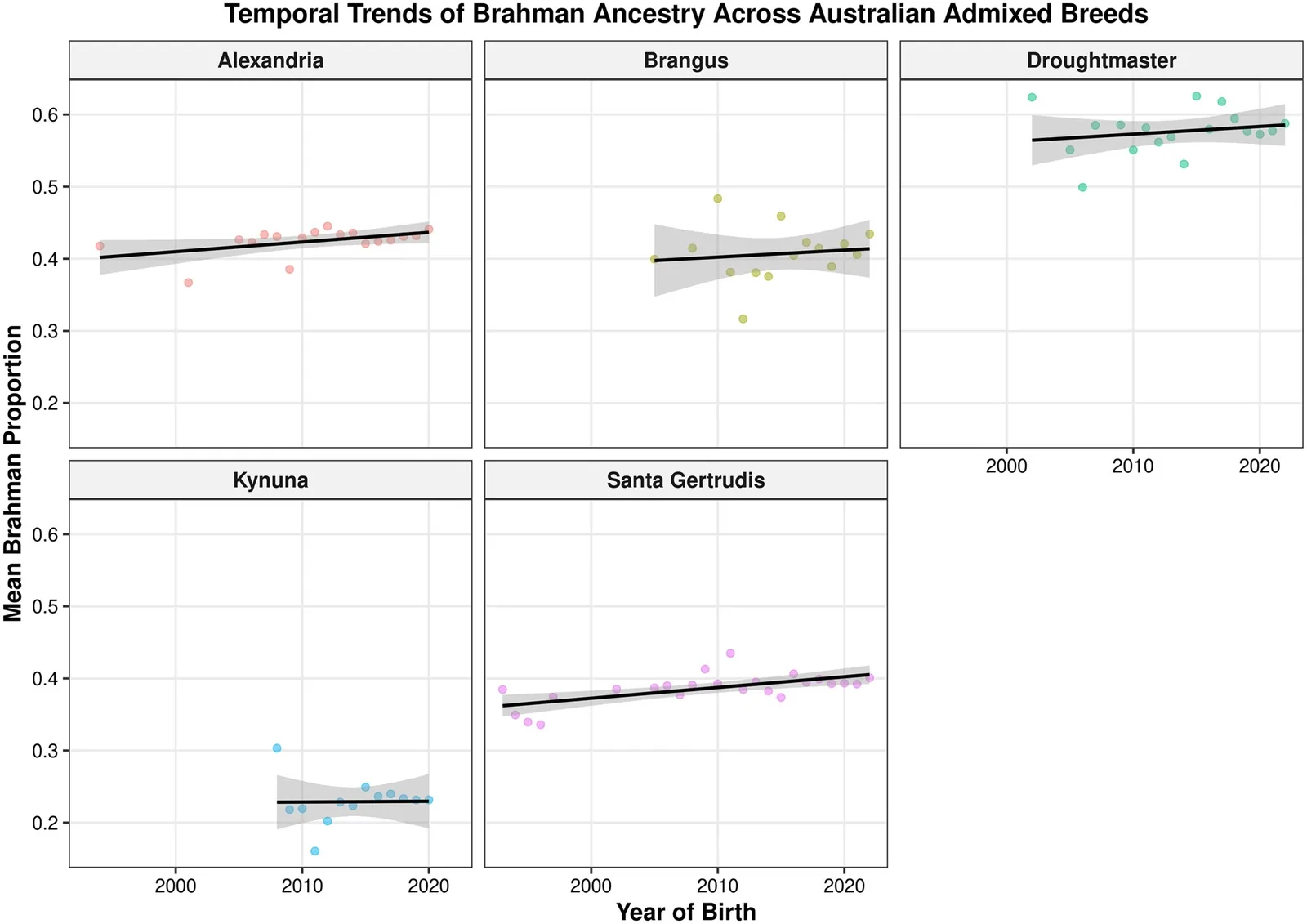
Temporal trends of Brahman ancestry across Australian admixed breeds estimated using supervised ADMIXTURE analysis with *K* = 7 reference ancestral populations.

A key finding of this study is the directional shift in ancestry proportions observed across the tropical composites over the years (Figure 5). A notable increase in the mean Brahman genomic component was identified in the Santa Gertrudis, Alexandria, and Droughtmaster populations. These findings provide support for the hypothesis of Hay et al. (2022), who argued that environmental selection pressure in tropical regions actively modulates the persistence of indicine alleles to maintain fitness, heat tolerance, and parasite resistance. Furthermore, this trend in Australian Brangus stands in direct contrast to the American Brangus (Paim et al. 2020), where a temporal shift toward the Angus progenitor was reported. The divergence between American and Australian Brangus trends suggests that local breeding objectives and environmental constraints play a definitive role in shaping their genome.

Although concerns are often raised regarding information loss when reducing SNP density, methodological evidence supports the robustness of our 8K common SNP panel. Boerner and Wittenburg (2018) demonstrated that accurate breed composition can be obtained even with 4K SNP panels using both genomic regression and ADMIXTURE approaches. In addition, Khorshidi (2022) observed that while higher-density panels (50K) improve individual accuracy, there is strict uniformity in estimated breed contributions between 6K and 50K panels when assessing population averages and autosomal segments.

### Limitations and future of population structure study of Australian beef cattle breeds

While this study provides a comprehensive genomic characterization of 13 Australian beef cattle breeds, several limitations remain that highlight opportunities for future research. A primary consideration is the potential for information loss when standardizing to an 8K common SNP panel. However, this density is robustly supported by Boichard et al. (2012), who demonstrated that low-density arrays (∼7K SNPs) can achieve imputation accuracies exceeding 97% across diverse beef and dairy populations. Furthermore, Boerner and Wittenburg (2018) and Khorshidi (2022) confirmed that such marker densities are sufficient for accurate ancestral inference and population structure. These findings validate that our 8K panel is an effective tool for population-level structure, even if it lacks the resolution for fine-scale genomic mapping.

Importantly, imputing the entire cross-breed dataset to high density (HD) was not feasible in this study. The accuracy of HD imputation is dependent on the size of breed-specific reference panels and the phylogenetic distance between populations. Given the significant genetic divergence between some of our 13 breeds, attempting a multi-breed imputation to HD would likely introduce substantial noise and reduce the reliability of genotypes. Future research, supported by larger shared reference populations and higher-density standardized panels, will be essential to resolve the fine-scale genomic features.

Further research should prioritize the implementation of advanced statistical techniques, such as weighted genotype likelihoods and breed-specific allele harmonization. These methodologies would enhance our ability to resolve precise ancestral contributions in complex crossbreeding histories, such as the Alexandria and Kynuna. Furthermore, there remains a clear opportunity to integrate these population structure findings into the industry-standard single-step genomic evaluation (ssGBLUP) procedures. Using the ancestry proportions identified here to define metafounders and construct accurate gamma matrices would improve the alignment between pedigree-based and GRMs. Such improvements are vital for enhancing the predictive accuracy of multi-breed genomic evaluations.

## Conclusions

In this study, PCA-complemented GMM was used for subsetting individuals in all breeds. The population structure analysis revealed clear genetic distinctions between *Bos indicus* and *Bos taurus* breeds. Admixed breeds exhibited varying genomic compositions due to contributions from multiple ancestral breeds. A major finding was the identification of directional shift in ancestry proportions within tropical composites, where a longitudinal increase in the Brahman genomic component indicates ongoing environmental adaptation to the Australian environment. These results confirm that Australian admixed breeds are dynamic entities responding to local breeding programs.

## Supplementary Material

skag242_Supplementary_Data

## Data Availability

The raw data is not available for public access since it is commercial and contains sensitive information.

## Abbreviations

BIC: Bayesian information criterion
CV: cross-validation
F_ST_: fixation index
GMM: Gaussian mixture model
GRM: genomic relationship matrix
HD: high density
IBS: identity by state
LD: linkage disequilibrium
MLH: multi-locus heterozygosity
NAPCo: North Australian Pastoral Company
NJ: neighbour-joining
NRM: pedigree-based relationship matrix
PCA: principal component analysis
SNP: single nucleotide polymorphism
ssGBLUP: single-step genomic BLUP
