# Supplementary material for: A genome-wide assessment of the population structure of thirteen admixed and pure Australian beef cattle breeds
Source: J Anim Sci. 2026 Aug 10;104:skag242. doi: 10.1093/jas/skag242 (PMC13537502; doi:10.1093/jas/skag242)
Supplement: Supplementary file 1 [file skag242_supplementary_data.docx]

Table S1 Weir and Cockerham F_ST_ values between the 260-animal subsets and full population datasets.

| **Pop1** | **Pop2** | **Weir and Cockerham F_ST_** |
| --- | --- | --- |
| **Alexandria_all** | **Alexandria_260** | 0.000208219 |
| **Angus_all** | **Angus_260** | 0.000197317 |
| **Brahman_all** | **Brahman_260** | 0.000200578 |
| **Brangus_all** | **Brangus_260** | 0.000156606 |
| **Charolais_all** | **Charolais_260** | -2.42745e-05 |
| **Droughtmaster_all** | **Droughtmaster_260** | 0.000550607 |
| **Hereford_all** | **Hereford_260** | 2.81961e-05 |
| **Kynuna_all** | **Kynuna_260** | 0.000367871 |
| **Limousin_all** | **Limousin_260** | -0.000114888 |
| **Santa Gertrudis_all** | **Santa Gertrudis_260** | 0.000398591 |
| **Shorthorn_all** | **Shorthorn_260** | -0.00136655 |
| **Speckle Park_all** | **Speckle Park_260** | 0.000187105 |
| **Wagyu_all** | **Wagyu_260** | 7.6173e-05 |

Table S2 Mean multi-locus heterozygosity (MLH) values across the 260-animal subsets and full populations using the 8K common SNP panel, and original genotypes in the full populations.

| Breed | 260 animals _ common SNPs | All animals _ common SNPs | All animals _ All SNPs |
| --- | --- | --- | --- |
| Alexandria | 0.427 ± 0.009 | 0.427 ± 0.010 | 0.38 ± 0.11 |
| Angus | 0.405 ± 0.014 | 0.406 ± 0.015 | 0.38 ± 0.11 |
| Brahman | 0.279 ± 0.024 | 0.277 ± 0.024 | 0.39 ± 0.11 |
| Brangus | 0.417 ± 0.017 | 0.417 ± 0.024 | 0.40 ± 0.10 |
| Charolais | 0.432 ± 0.017 | 0.434 ± 0.013 | 0.40 ± 0.09 |
| Droughtmaster | 0.405 ± 0.013 | 0.402 ± 0.015 | 0.42 ± 0.09 |
| Hereford | 0.401 ± 0.018 | 0.401 ± 0.015 | 0.39 ± 0.10 |
| Kynuna | 0.439 ± 0.010 | 0.438 ± 0.010 | 0.38 ± 0.11 |
| Limousin | 0.426 ± 0.012 | 0.425 ± 0.013 | 0.40 ± 0.09 |
| Santa Gertrudis | 0.400 ± 0.013 | 0.401 ± 0.014 | 0.37 ± 0.11 |
| Shorthorn | 0.403 ± 0.013 | 0.403 ± 0.013 | 0.37 ± 0.10 |
| Speckle Park | 0.406 ± 0.016 | 0.406 ± 0.016 | 0.40 ± 0.10 |
| Wagyu | 0.351 ± 0.025 | 0.348 ± 0.021 | 0.36 ± 0.11 |

Table S3. Validation of pedigree diversity for the 260-animal subsets and full populations across all thirteen breeds.

| **Breed** | **All Animals** | **Subset_Size** | **Matched_Subset_Size** | **Top10_Genotyped_Perc** | **Top10_Subset_Perc** | **Unique_Sires_Subset** | **Offspring_Per_Sire** |
| --- | --- | --- | --- | --- | --- | --- | --- |
| **aa** | 289,460 | 260 | 259 | 8.13 | 16.22 | 200 | 1.29 |
| **bb** | 48,375 | 260 | 260 | 3.8 | 5.77 | 193 | 1.35 |
| **bg** | 8,226 | 260 | 260 | 11.12 | 14.23 | 160 | 1.62 |
| **cc** | 5,711 | 260 | 260 | 9.65 | 11.15 | 186 | 1.4 |
| **dm** | 7,674 | 260 | 260 | 12.67 | 31.92 | 121 | 2.15 |
| **hh** | 59,387 | 260 | 260 | 5.46 | 7.69 | 222 | 1.17 |
| **ll** | 3,810 | 260 | 260 | 11.36 | 11.54 | 173 | 1.5 |
| **sg** | 8,141 | 260 | 257 | 12.19 | 26.07 | 139 | 1.85 |
| **sk** | 13,143 | 260 | 260 | 17.26 | 16.92 | 156 | 1.67 |
| **xa** | 11,553 | 260 | 260 | 10.36 | 21.92 | 125 | 2.08 |
| **xk** | 4,087 | 260 | 212 | 13.9 | 28.3 | 105 | 2.02 |

All Animals: Total number of genotyped individuals in the full population for each breed. Matched Subset Size: Actual number of animals in the final subset successfully matched with pedigree information. Top10_Genotyped_Perc: Percentage of the full genotyped population sired by the ten most influential bulls. Top10_Subset_Perc: Percentage of the 260-animal subset sired by the ten most influential bulls. Unique Sires Subset: Total count of unique sires represented within the 260-animal subset. Offspring Per Sire: Average number of progenies per sire in the 260-animal subset. Abbreviations: aa, Angus; bb, Brahman; bg, Brangus; cc, Charolais; dm, Droughtmaster; hh, Hereford; ll, Limousin; sg, Santa Gertrudis; sk, Speckle Park; ss, Shorthorn; xa, Alexandria; xk, Kynuna.

Figure S1 A diagram of the breed composition of Alexandria and Kynuna breeds: https://www.beefcentral.com/genetics/weekly-genetics-review-pastoral-companies-big-on-composite-breeding-programs.

Figure S2 Distribution of genotyped animals per year of birth from 1990 to 2022.

Figure S3 Density-PCA plot of first and second principal components from thirteen beef cattle breeds. Abbreviations: aa, Angus; bb, Brahman; bg, Brangus; cc, Charolais; dm, Droughtmaster; hh, Hereford; ll, Limousin; sg, Santa Gertrudis; sk, Speckle Park; ss, Shorthorn; wy, Wagyu; xa, Alexandria; xk, Kynuna.

Figure S4 Plots explaining variance and eigenvalues for the twenty Principal Components (PCs) from the PCA analysis. The red line represents 50% variance. Abbreviations: aa, Angus; bb, Brahman; bg, Brangus; cc, Charolais; dm, Droughtmaster; hh, Hereford; ll, Limousin; sg, Santa Gertrudis; sk, Speckle Park; ss, Shorthorn; wy, Wagyu; xa, Alexandria; xk, Kynuna.

Figure S5 Bayesian Information Criterion Values for detecting the optimal cluster number in each breed based on Gaussian Mixture Models (GMM). The breed abbreviations are defined in Table 3.1. In the plot, 14 models were examined to find the optimal number of cluster, including "EII" = spherical, equal volume "VII" = spherical, unequal volume, "EEI" = diagonal, equal volume and shape, "VEI" = diagonal, varying volume, equal shape, "EVI" = diagonal, equal volume, varying shape, "VVI" = diagonal, varying volume and shape, "EEE" = ellipsoidal, equal volume, shape, and orientation, "EVE" = ellipsoidal, equal volume and orientation, "VEE" = ellipsoidal, equal shape and orientation, "VVE" = ellipsoidal, equal orientation, "EEV" = ellipsoidal, equal volume and equal shape, "VEV" = ellipsoidal, equal shape, "EVV" = ellipsoidal, equal volume, "VVV" = ellipsoidal, varying volume, shape, and orientation.

Figure S6 Phylogenetic relationships for thirteen Australian beef cattle breeds with actual sample size. The heatmap and dendrogram of pairwise fixation index (FST). Abbreviations: aa, Angus; bb, Brahman; bg, Brangus; cc, Charolais; dm, Droughtmaster; hh, Hereford; ll, Limousin; sg, Santa Gertrudis; sk, Speckle Park; ss, Shorthorn; wy, Wagyu; xa, Alexandria; xk, Kynuna.

Figure S7 Principal Component Analysis (PCA) across breeds, conducted in three independent replicates.

Figure S8 Supervised ADMIXTURE analysis across breeds conducted in two independent replicates. Abbreviations: aa, Angus; bb, Brahman; bg, Brangus; cc, Charolais; dm, Droughtmaster; hh, Hereford; ll, Limousin; sg, Santa Gertrudis; sk, Speckle Park; ss, Shorthorn; wy, Wagyu; xa, Alexandria; xk, Kynuna.

Figure S9 Linkage disequilibrium (LD) decay across the 8K common SNP panel by breed.

Figure S10 Phylogenetic relationship of the pairwise identity-by-state (IBS) distance for thirteen Australian beef cattle breeds. Abbreviations: aa, Angus; bb, Brahman; bg, Brangus; cc, Charolais; dm, Droughtmaster; hh, Hereford; ll, Limousin; sg, Santa Gertrudis; sk, Speckle Park; ss, Shorthorn; wy, Wagyu; xa, Alexandria; xk, Kynuna.

Figure S11 Cross-validation error plot of unsupervised ADMIXTURE analysis. Y-axis represents cross-validation errors and X-axis represents ancestral clusters (K) values from 1-20.
